# Impact of Lianhua Qingwen on viral shedding in omicron mild/asymtomatic patients: a real-world study

**DOI:** 10.3389/fmed.2024.1357299

**Published:** 2024-03-26

**Authors:** Xiaoyan Gai, Changjian Yan, Chaoling Wu, Zhonghui Duan, Jiarun Fan, Shengren Yuan, Ping Yang, Fang Bao, Hongmei Jing, Chao Cai, Yingmin Ma, Weilong Zhang, Yongchang Sun

**Affiliations:** ^1^Department of Respiratory and Critical Care Medicine, Peking University Third Hospital, and Center for Chronic Airway Diseases, Peking University Health Science Center, Peking University, Beijing, China; ^2^Department of Hematology, Lymphoma Research Center, Peking University Third Hospital, Beijing, China; ^3^Department of Respiratory and Critical Care Medicine, Beijing Youan Hospital, Capital Medical University, Beijing, China

**Keywords:** Lianhua Qingwen, LHQW, SARS-CoV-2, COVID-19, omicron, real-world study

## Abstract

**Background:**

Lianhuaqingwen (LHQW), a traditional Chinese medicine comprised of 13 herbal extracts renowned for their robust heat-clearing and detoxifying properties, has gained widespread utilization in China but has yet to garner similar recognition abroad. It is believed to exhibit efficacy in ameliorating symptoms in individuals afflicted with coronavirus disease 2019 (COVID-19). However, the precise impact of LHQW on viral shedding (VS), particularly in the context of mild or asymptomatic infections caused by the Omicron BF.4/5 or BF.7 variants of COVID-19, remained inadequately elucidated. Consequently, a real-world study was conducted, involving patients diagnosed with COVID-19, with the primary objective of ascertaining the effectiveness of LHQW in this specific clinical context.

**Methods:**

We conducted an investigation on Omicron-infected patients through a single-center, propensity score-matched real-world study conducted at Xiaotangshan Fangcang Hospital from May to November 2022. A total of 3,368 COVID-19 patients were enrolled in the study, all of whom presented mild or asymptomatic infections caused by either BF.4/5 or BF.7 strains of the virus. Demographic and clinical data were systematically collected from medical records. Patients were allocated to receive treatment with LHQW (designated as the treatment group) or received no LHQW treatment (designated as the not-treated/no-treatment group). Viral load was quantified utilizing quantitative real-time PCR (qPCR), and the duration of VS was defined as the time interval between the initial negative test result and the date of COVID-19 diagnosis or symptom onset.

**Results:**

The study encompassed a cohort of 3,368 patients, and following propensity score matching, a subset of 296 patients was meticulously chosen for subsequent analysis. Notably, baseline characteristics exhibited disparities between the treatment and not-treated/no-treatment groups. However, post-matching, these characteristics achieved a commendable level of comparability. Our findings unequivocally demonstrated that there existed no statistically significant disparity in VS. This holds true when comparing patients subjected to LHQW treatment against those not administered LHQW, as well as when contrasting individuals presenting asymptomatic and mild COVID-19 manifestations.

**Conclusion:**

No statistically significant difference in VS was observed between patients who underwent LHQW treatment and those who did not. Additional investigations are imperative to provide a comprehensive assessment of LHQW’s efficacy, particularly in patients afflicted with severe COVID-19 or those infected with viral strains distinct from BF.4/5 or BF.7.

## Introduction

COVID-19, precipitated by the SARS-CoV-2 virus, has disseminated on a global scale since late 2019, presenting formidable healthcare challenges. The disease manifests with symptoms such as fever, cough, respiratory distress, anosmia, and ageusia, and in severe instances, it can prove fatal (1, 2). The Omicron variant of the SARS-CoV-2 virus was first identified in November 2021 in Botswana and South Africa (3). Subsequently, Omicron variants BF.7 and BA.4/5 emerged as the predominant strains. These variants exhibit numerous mutations in their spike protein, which could potentially enhance transmissibility, confer resistance to antibodies, or lead to milder symptomatology when compared to earlier strains. Following infection, symptoms may endure until the virus is cleared from the body (4). Individuals with preexisting medical conditions are at an elevated risk of experiencing severe illness and life-threatening complications subsequent to contracting the Omicron variant of the virus (5).

Viral load and respiratory testing of SARS-CoV-2 are key to estimating infectivity, and shedding of infectious virus is necessary for continued transmission (6), therefore, VS is closely related to infectivity. SARS-CoV-2 can be transmitted in a variety of ways, including through the excrement of a person with COVID-19 into wastewater. Recent research has identified that SARS-CoV-2 virus strains in fecal water are constantly mutating and dominated by co-circulating variants (7). Many drugs can reduce VS, but there are not enough studies on them, so it is important to study the relationship between LHQW and VS.

Currently, supportive therapies stand as the cornerstone of COVID-19 management. Traditional Chinese medicine, owing to its extensive historical use and proven effectiveness in influenza patients (8), has been recently adapted for clinical COVID-19 management (9). Numerous potential antiviral agents have been subject to investigation (10). Among these, the widely employed Lianhuaqingwen (LHQW) capsule, developed by Shijiazhuang Yiling Pharmaceutical Co. Ltd. in Shijiazhuang, China, contains 13 herbal extracts with robust heat-clearing and detoxifying properties, thereby demonstrating effectiveness in mitigating lung inflammation. A prospective, multicenter, randomized controlled trial was conducted across 23 hospitals spanning 9 provinces in China to elucidate the potential of LHQW in ameliorating clinical symptoms such as fever, fatigue, and cough, while concurrently improving chest imaging and reducing symptom duration and expediting recovery time (11). *In vitro* experiments provided compelling evidence supporting LHQW’s anti-SARS-CoV-2 efficacy. LHQW was reported to demonstrate antiviral activity by inhibiting SARS-CoV-2 replication, suppressing the production of pro-inflammatory cytokines (TNF-α, IL-6, MCP-1, and IP-10), and influencing virion morphology (12). Previous research indicated that LHQW has shown effectiveness in treating infections caused by the Omicron strain of the novel coronavirus, with some studies suggesting its potential to facilitate viral clearance (11, 13–17). Nevertheless, there is a scarcity of relevant investigations regarding its influence on VS. The present study is constrained by an insufficient sample size and the absence of real-world studies in this context.

This study endeavors to assess and compare the therapeutic efficacy between the LHQW treatment group and the LHQW not-treated/no-treatment group in patients afflicted with the Omicron variant through real-world observations. Our findings offer a novel perspective on the utilization of LHQW in the context of COVID-19 treatment.

## Methods and materials

### Participant recruitment and data acquisition

**Figure 1:**
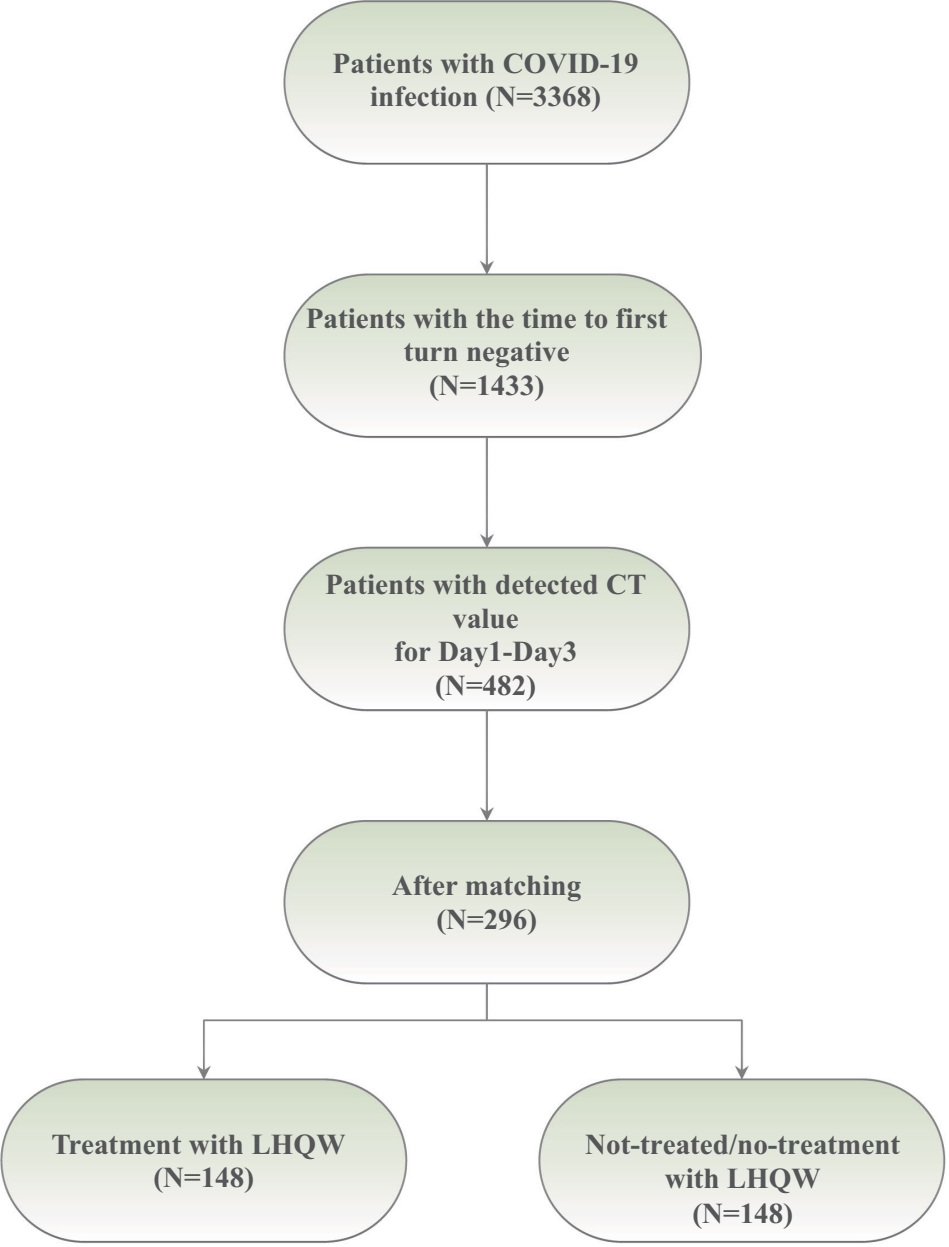
A summary workflow of the present study.

This research was conducted at Xiaotangshan Fangcang Hospital, located in Beijing, China, in strict accordance with the ethical principles delineated in the Declaration of Helsinki. The study protocol received approval from the Ethics Committee at Peking University Third Hospital. Participants were enrolled at Fangcang Hospital during two distinct time intervals: from May to June 2022 (for BA.4/5) and from October to November 2022 (for BF.7). In total, 3,368 patients were recruited for the study. The inclusion criteria encompassed individuals who tested positive for COVID-19 via nucleic acid testing, comprising asymptomatic infections (those diagnosed with COVID-19 but devoid of clinical symptoms) and mild patients (individuals exhibiting minor symptoms without pneumonia evident in medical imaging). Age was not a limiting factor. Exclusion criteria were defined to exclude patients in critical condition, those incapable of completing the nucleic acid test, or those who were transferred out of Fangcang Hospital during the study period. Population demographics and clinical characteristics were meticulously extracted from electronic health records. The severity of COVID-19 was categorized into two groups, namely asymptomatic infections and mild patients. We initially screened 482 patients with CT values recorded for Day 1 to Day 3, and subsequent to propensity score matching (PSM), a cohort of 296 patients was ultimately selected (Figure 1A).

### Quantification of viral load through qPCR analysis

We diligently monitored VS at multiple, closely spaced time points. Nucleic acid testing was performed sequentially, with an average of 7 tests conducted per patient. The quantitative real-time PCR (qPCR) assay specifically targeted the open reading frame lab (ORFlab) and the nucleocapsid protein (N) as the designated target genes. Nasopharyngeal swab samples were processed following the instructions provided by the Marburg virus nucleic acid detection kit from Shanghai BioGerm Medical Technology Co., Ltd. (CHN). Viral RNA was efficiently extracted and detected from these samples. Viral load was quantified using qPCR and was denoted by the cycle threshold (CT) value. The CT cut-off threshold for determining the presence of VS was set at 35. If the CT values for both N and ORFlab were equal to or greater than 35, it was deemed a negative result; otherwise, it was classified as a positive result. In instances where a patient’s CT value exceeded 35, a follow-up nucleic acid test was scheduled to confirm a negative result on the subsequent day. Conversely, if the CT value remained below 35, daily nucleic acid testing was continued until a CT value exceeding 35 was obtained, subsequently leading to the confirmation of a negative result on the following day. The duration of viral shedding (VS) was calculated as the time interval between the date of the initial negative result and the date of COVID-19 diagnosis or symptom onset.

## Results

The baseline demographic and clinical characteristics, both before and after propensity score matching (PSM), have been comprehensively summarized in Table 1. Following PSM, no statistically significant differences were observed between the LHQW-treated and LHQW-untreated groups. In both groups, a higher prevalence of male patients was noted compared to female patients. Furthermore, cases of asymptomatic infection exceeded those of mild infection in both cohorts. Additionally, a majority of patients in both groups did not report habits of smoking or alcohol consumption, and a higher proportion of patients were devoid of comorbidities such as hypertension, fever, and allergies. At the baseline, there were no statistically significant differences in CT values for the ORF1ab gene and N gene between the two groups. Moreover, the majority of patients did not receive alternative treatments such as Qinggan, Paxlovid, and Azvudine; however, it is worth noting that the distinction between the LHQW-treated and LHQW-not-treated/no-treatment groups in this regard was not statistically significant.

**Table 1:** Baseline characteristics of COVID19 patients treated with LHQW and untreated with LHQW before and after PSM.

Characteristics	Level	Before PSM cohort (*N* = 482)			*p*	After PSM cohort (*N* = 296)			*p*
		Overall	Treatment	Not-treated/no-treatment		Overall	Treatment	Not-treated/no-treatment	
*n*		482	291	191		296	148	148	
Group (%)	BA.4/5	280 (58.1)	170 (58.4)	110 (57.6)	0.932	175 (59.1)	86 (58.1)	89 (60.1)	0.813
	BF.7	202 (41.9)	121 (41.6)	81 (42.4)		121 (40.9)	62 (41.9)	59 (39.9)	
Age [mean (SD)]		31.46 (13.50)	30.67 (12.93)	32.66 (14.28)	0.113	31.09 (12.89)	31.32 (12.87)	30.86 (12.96)	0.763
Sex (%)	Female	211 (43.8)	118 (40.5)	93 (48.7)	0.095	125 (42.2)	60 (40.5)	65 (43.9)	0.638
	Male	271 (56.2)	173 (59.5)	98 (51.3)		171 (57.8)	88 (59.5)	83 (56.1)	
Diagnose (%)	Asymptomatic infection	316 (65.6)	190 (65.3)	126 (66.0)	0.956	189 (63.9)	91 (61.5)	98 (66.2)	0.468
	Mild	166 (34.4)	101 (34.7)	65 (34.0)		107 (36.1)	57 (38.5)	50 (33.8)	
Alcohol (%)	No	435 (90.2)	261 (89.7)	174 (91.1)	0.724	272 (91.9)	136 (91.9)	136 (91.9)	1
	Yes	47 (9.8)	30 (10.3)	17 (8.9)		24 (8.1)	12 (8.1)	12 (8.1)	
Smoke (%)	No	429 (89.0)	255 (87.6)	174 (91.1)	0.297	275 (92.9)	139 (93.9)	136 (91.9)	0.651
	Yes	53 (11.0)	36 (12.4)	17 (8.9)		21 (7.1)	9 (6.1)	12 (8.1)	
HP (%)	No	478 (99.2)	288 (99.0)	190 (99.5)	0.93	295 (99.7)	147 (99.3)	148 (100.0)	1
	Yes	4 (0.8)	3 (1.0)	1 (0.5)		1 (0.3)	1 (0.7)	0 (0.0)	
Fever (%)	No	303 (62.9)	163 (56.0)	140 (73.3)	<0.001	215 (72.6)	107 (72.3)	108 (73.0)	1
	Yes	179 (37.1)	128 (44.0)	51 (26.7)		81 (27.4)	41 (27.7)	40 (27.0)	
Allergy (%)	No	463 (96.1)	285 (97.9)	178 (93.2)	0.017	289 (97.6)	144 (97.3)	145 (98.0)	1
	Yes	19 (3.9)	6 (2.1)	13 (6.8)		7 (2.4)	4 (2.7)	3 (2.0)	
CT3minOR [mean (SD)]		22.28 (4.98)	22.22 (4.58)	22.38 (5.56)	0.723	22.30 (5.19)	22.52 (4.80)	22.08 (5.56)	0.461
CT3minN [mean (SD)]		20.76 (5.36)	20.68 (5.01)	20.88 (5.87)	0.683	20.77 (5.58)	20.97 (5.30)	20.58 (5.86)	0.548
Qinggan (%)	No	401 (83.2)	238 (81.8)	163 (85.3)	0.37	246 (83.11)	120 (81.08)	126 (85.14)	0.438
	Yes	81 (16.8)	53 (18.2)	28 (14.7)		50 (16.89)	28 (18.92)	22 (14.86)	
Paxlovid (%)	No	471 (97.7)	281 (96.6)	190 (99.5)	0.075	289 (97.64)	142 (95.95)	147 (99.32)	0.126
	Yes	11 (2.3)	10 (3.4)	1 (0.5)		7 (2.36)	6 (4.05)	1 (0.68)	
Azvudine (%)	No	474 (98.3)	285 (97.9)	189 (99.0)	0.625	295 (99.66)	148 (100.00)	147 (99.32)	1
	Yes	8 (1.7)	6 (2.1)	2 (1.0)		1 (0.34)	0 (0.00)	1 (0.68)	

**Figure 2:**
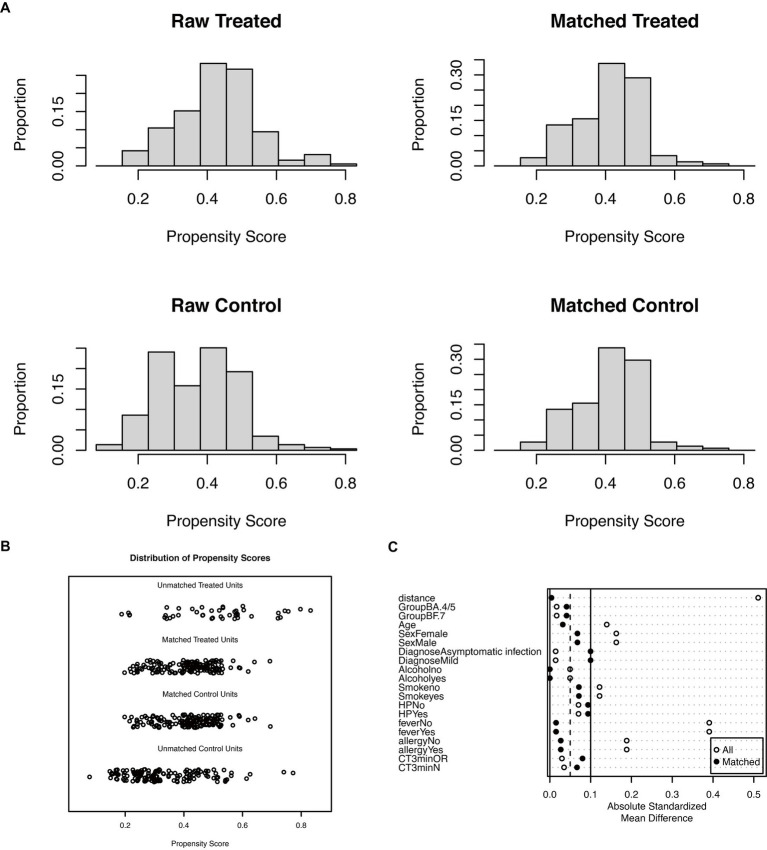
Propensity score before and after matching. **(A)** Histogram for propensity score of the raw and matched group. **(B)** Dotplot for propensity score of the raw and matched group. **(C)** Dotplot of the absolute standardized mean difference.

**Figure 3:**
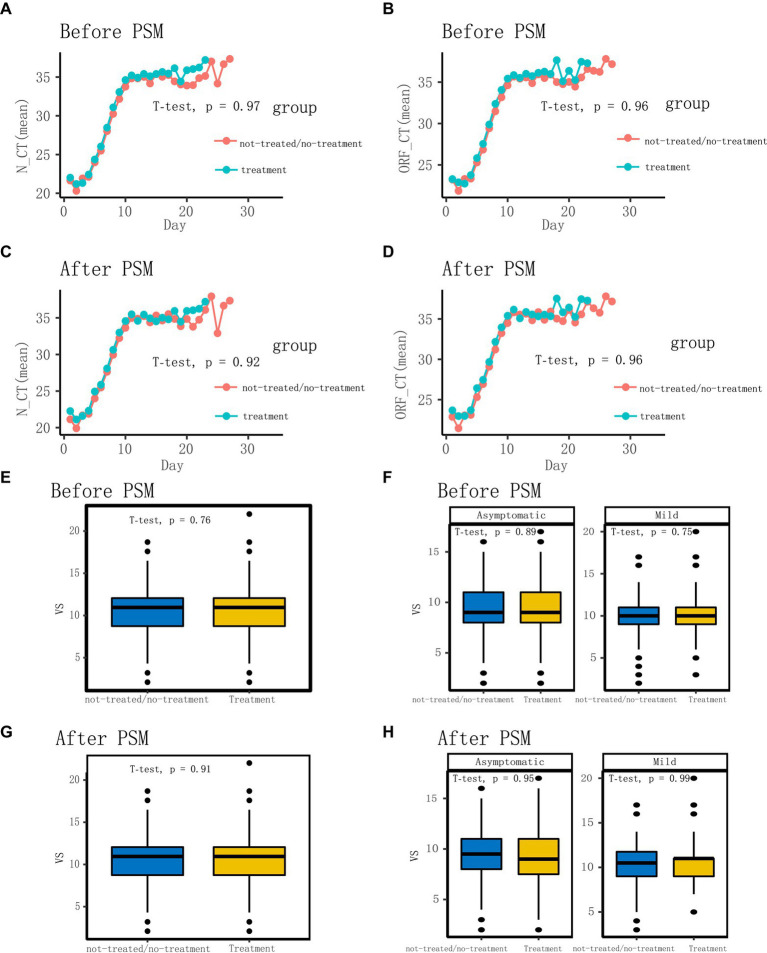
Comparison of characteristics before and after matching. **(A)** Comparison of N-CT (mean) between not-treated/no-treatment and treatment groups before matching. **(B)** Comparison of ORF-CT (mean) between not-treated/no-treatment and treatment groups before matching. **(C)** Comparison of N-CT (mean) between not-treated/no-treatment and treatment groups after matching. **(D)** Comparison of ORF-CT (mean) between not-treated/no-treatment and treatment groups after matching. **(E,F)** Comparison of VS between not-treated/no-treatment and treatment groups before matching. **(G,H)** Comparison of VS between not-treated/no-treatment and treatment groups after matching.

Following the application of the PSM matching technique to balance the baseline characteristics of the two groups, we identified and selected 296 patients in a 1:1 ratio, comprising 148 patients in each of the LHQW-treated and LHQW-not-treated/no-treatment groups (Figure 1A). Upon scrutinizing the characteristics of both groups after matching, it was observed that the propensity score distribution of the two groups was concordant, and the matching score effect exhibited stability and reliability. This observation suggested that the data from both groups post-matching was more comparable (Figure 2A). In light of this, we conducted a thorough comparison of the disparities in baseline characteristics between the two patient groups following matching. The outcomes revealed no statistically significant differences in baseline characteristics between the two groups (Figure 2B). To further illustrate the effectiveness of the matching process, Figure 2C presents a visualization of the standardized mean difference (SMD) both before and after matching, underscoring the reduction in SMD achieved through the matching procedure. In the subsequent analysis, after matching, it was discerned that the LHQW-treated group and LHQW-not-treated/no-treatment group displayed analogous trends in terms of both N-CT and ORF-CT (Figures 3A–D). Furthermore, the investigation unveiled that there was no significant disparity in VS between the two groups, both among individuals with asymptomatic and mild infections (Figures 3E–H).

## Discussion

The primary objective of this study was to conduct a comparative analysis of VS in COVID-19 patients who underwent LHQW treatment versus those who did not receive any treatment. Our study encompassed a substantial sample size comprising 3,368 patients afflicted with either the Omicron BF.7 or Omicron BA.4/5 variants, thereby ensuring the availability of robust and representative data for our analysis.

Prior research in the field has predominantly concentrated on assessing the effectiveness of LHQW in ameliorating clinical symptoms, with limited insights into its impact on VS (11–13, 15, 16, 18, 19). For instance, Academician Zhong’s recent publication reports findings from an international, multicenter, double-blind, randomized controlled study, primarily focusing on the alleviation of COVID-19 symptoms by LHQW, encompassing nasal congestion, sore throat, cough, fever, and other related symptoms (20). The results indicate that LHQW can significantly and expeditiously reduce the relief time and enhance the recovery rate of major COVID-19 symptoms. However, it is noteworthy that the mentioned study did not address the VS. Consequently, our research serves as a complementary study to Academician Zhong’s investigation into LHQW, addressing this specific aspect that was not covered in his study. A prospective, multicenter, randomized controlled trial spanning 23 hospitals across 9 provinces in China demonstrated the capacity of LHQW to alleviate clinical symptoms such as fever, malaise, and cough, while also leading to improvements in chest radiographic outcomes. However, it did not yield significant improvements in the conversion rate of viral assays (11), a result in line with our own findings. It’s noteworthy that their research pertained to the early SARS-CoV-2 strain, whereas our investigation centered on the Omicron variant, a subject they did not explore. Nevertheless, it’s worth noting that there are also conflicting studies in the literature that diverge from our results. A study involving children, with a total of 692 samples, revealed that the LHQW treatment group exhibited a reduction in the time required for VS (21). This suggested that LHQW might be efficacious in expediting reduction of VS in pediatric patients. We believed that our study came to the opposite conclusion for two main reasons, Firstly, the virus under investigation in the referenced study was the Omicron BF.2 variant, while our research focused on the Omicron BF.4/5 or BF.7 variants. Secondly, the demographic of the referenced study comprised children, in contrast to our study, which targeted the adult population. These distinctions in both the viral variants and the study populations were pivotal in understanding the differing outcomes observed. Conversely, another study encompassing a large adult population of 4,918 cases demonstrated that the LHQW treatment group exhibited improvements in the nucleic acid conversion rate (19). Numerous systematic reviews and meta-analyses have underscored the substantial efficacy of LHQW in the treatment of COVID-19 (14, 15, 22), reporting enhancements in clinical symptoms and a reduction in the progression to severe or critical conditions, but they did not specifically monitor VS. The most notable difference between our study and these prior investigations lies in the diverse endpoints used to evaluate LHQW efficacy, as well as the distinct populations of interest under study. For patients battling severe COVID-19, there is an unequivocal imperative to prioritize the amelioration of symptoms and prognosis. Conversely, in the case of patients experiencing mild or even asymptomatic COVID-19, the emphasis on symptom improvement becomes less pertinent. In such instances, directing our focus toward VS, as conducted in our study, assumes paramount significance. Furthermore, a study conducted by Fang et al. reported a synergistic effect between LHQW and dexamethasone in expediting the attainment of nucleic acid negativity in patients afflicted with severe COVID-19 (23). However, it’s crucial to acknowledge that the quantity and quality of the studies included in this line of research remain limited. Hence, the necessity for additional high-quality clinical trials incorporating additional observational indicators is evident to substantiate these findings (18, 23, 24).

This study is subject to several limitations. Firstly, it is important to acknowledge that this is a retrospective study rather than a prospective one, potentially affecting the robustness of our findings. Secondly, our study exclusively enrolled patients with mild and asymptomatic COVID-19 infections caused by the BA.4/5 or BF.7 strains. Consequently, the efficacy of LHQW in patients with severe COVID-19 or those infected with strains distinct from BA.4/5 or BF.7 remains uncharted territory and warrants further investigation.

In summary, our study has unveiled that there exists no substantial discrepancy in VS between the cohort of patients subjected to LHQW treatment and the not-treated/no-treatment group. This observation introduces a novel dimension to the discourse surrounding the utility of LHQW in the management of COVID-19. It underscores the necessity for additional research endeavors aimed at providing a comprehensive evaluation of its efficacy and feasibility in clinical practice. Furthermore, future cohorts should be expanded to encompass patients grappling with severe COVID-19 manifestations or those infected with viral strains divergent from BF.4/5 or BF.7. Such investigations will contribute to a more comprehensive understanding of the potential enhancements in VS attributed to LHQW in these distinct clinical contexts.

## Data availability statement

The original contributions presented in the study are included in the article/supplementary material, further inquiries can be directed to the corresponding authors.

## Ethics statement

The studies involving humans were approved by the Ethics Committee of Peking University Third Hospital. The studies were conducted in accordance with the local legislation and institutional requirements. Given the urgent demand for epidemiological and clinical data acquisition and the retrospective nature of our study, the Ethics Committees granted a waiver of prior informed consent. All data were meticulously analyzed in an anonymous fashion to safeguard the privacy of participants.

## Author contributions

XG: Writing – review & editing, Writing – original draft, Validation, Supervision, Resources, Project administration, Methodology, Investigation, Funding acquisition, Data curation, Conceptualization. CY: Writing – review & editing, Writing – original draft, Validation, Resources, Investigation, Data curation, Conceptualization. CW: Writing – review & editing, Writing – original draft, Validation, Resources, Investigation, Data curation, Conceptualization. ZD: Writing – review & editing, Writing – original draft, Validation, Investigation, Data curation. JF: Writing – review & editing, Writing – original draft, Validation, Investigation, Data curation. SY: Writing – review & editing, Writing – original draft, Validation, Investigation, Data curation. PY: Writing – review & editing, Writing – original draft, Validation, Investigation, Data curation. FB: Writing – review & editing, Writing – original draft, Validation, Investigation, Data curation. HJ: Writing – review & editing, Writing – original draft, Validation, Investigation, Funding acquisition, Data curation. CC: Writing – review & editing, Writing – original draft, Validation, Investigation, Data curation. YM: Writing – review & editing, Writing – original draft, Validation, Investigation, Data curation. WZ: Writing – review & editing, Writing – original draft, Validation, Supervision, Software, Resources, Project administration, Methodology, Investigation, Funding acquisition, Formal analysis, Data curation, Conceptualization. YS: Writing – review & editing, Writing – original draft, Validation, Supervision, Software, Resources, Project administration, Methodology, Investigation, Formal analysis, Data curation, Conceptualization.
